# On Bronchotomy in Œdematous Laryngeal Angina

**Published:** 1851-10

**Authors:** 


### SURGERY.

*On Bronchotomy in Œdematous Laryngeal Angina.* By M. SESTIER.—This is a very elaborate and valuable paper, having for its object, the inculcation of a more frequent recourse to operative procedures for the relief of œdematous laryngeal angina, commonly called œdema of the glottis; a term objected to by Dr. Sestier, inasmuch as the arytenoid-epiglotteal folds are much oftener the seat of the disease than the rima glottidis. The author bases his remarks upon 168 cases of the disease which he has collected, in 36 of which the operation was performed often under very unfavorable circumstances, saving life in 13 and prolonging it in 8, although death eventually occurred. He minutely analyses the circumstances under which the disease occurred, and exhibits the amount of success to be expected from the operation, accordingly as it is applied to the different categories into which he has distributed the cases. He also compares the results obtainable from it in this and in other affections; and finds that, while the operation for œdema is less successful than when undertaken for erythematous laryngitis, or for the removal of foreign bodies, it is more so than when performed for croup.

From his investigations it results, that the probability of its success is very much dependent upon the prior healthy state of the larynx, and the fact of the patient not suffering from other serious disease. But he believes it should still be performed even when the œdema is consecutive to severe laryngeal disease, though it has then succeeded only in 1 out of 19 cases, during convalescence from other disease though it has failed in 4 out of 6 such cases, and when concomitant with various other diseases, though it has then failed in 15 out of 17 times. In fact, he would recommend it in all cases, save when the angina is the *ultimate phenomenon* of incurable and advanced disease, the fatal issue of which would be probably accelerated by it. In several such cases, however, the object would be only to prolong life, and this should be distinctly stated; but in others, apparently desperate, nature's resources would be by it brought advantageously into play.

As to the *epoch* at which the operation should be undertaken, this must *not* be as soon as the disease is recognized, for in 28 well-marked cases it was cured by ordinary means; and these, when energetic, have several times proved of avail even after severe suffocative paroxysms and great intervening dyspnœa have become established. On the other hand, we must *not wait* until it is absolutely certain that the patient will speedily die unless the operation be performed, as he would then often die during or soon after it. We must not be deceived by the dangerous calm which follows prolonged suffocative paroxysm, and is only the precursor of speedy death. It is difficult to fix any precise period; but it may be laid down, that if in spite of active means rapidly employed, the difficulty of respiration continues to increase, *the respiratory murmur heard by auscultation becomes more and more feeble,* and the suffocative paroxysms are on the increase, the operation is urgently indicated; and it is far more safe to operate *too soon* than *too late,* success being proportioned to the early performance.

There are four circumstances under which the performance of the operation should be hastened. 1. *The debility of the patient at the period of the invasion of the angina;* and doubtless this is the reason why it has so frequently failed, when had recourse to during convalescence from other diseases. The more enfeebled the patient prior to the invasion, the earlier should be the operation. 2. The presence of *deep-seated lesions of the larynx* prior to the invasion. As these have induced the œdema, so they will maintain it. 3. *Œdema of the interior of the larynx.* In 41 autopsies out of 107 collected by the author, partial intra-laryngeal œdema prevailed in 18, and complete in 23, the *chordæ vocales* being almost always implicated in both. In four-sevenths of 57 cases of laryngeal œdema, in which the interior of the larynx has been described, this has been present. It adds much to the danger of the case, inasmuch as it is inaccessible to direct applications, and the close texture of the cellular tissue here causes the disease to yield less readily to indirect ones. Its diagnosis is therefore important, and is derived from observing (1,) that it never occurs but in patients who were already ill from some cause when the œdema appeared; (2,) when found in œdematous angina in connection with inflammation of the fauces, the patients have already suffered from some other forms of disease, and especially from serous diatheses; (3,) it has been found in three-sevenths of the cases of œdematous angina dependent upon laryngitis, and especially when the serous diathesis was present; (4,) the facility of *expiration,* as contrasted with that of *inspiration,* characteristic of ordinary œdematous laryngeal angina, was not observed in seven-twelfths of the cases in which intra-laryngeal œdema was present—the obstacle to respiration being then a more fixed one; (5,) in 11 out of 12 cases of laryngeal œdema in which the fauces were found infiltrated, the œdema was also intra-laryngeal. 4. *Rapidly increasing œdema of the soft parts of the neck,* which renders the operation difficult or impossible.

M. Sestier recommends an operation, even if the patient seems in the *agony of death,* providing this depends upon the œdema itself, and not upon preceding irremediable disease. Cases are related of life being thus saved. Even when the patient is *apparently dead,* we must not always renounce the operation. A recovery under these circumstances occurred to M. Trousseau; and some of the patients who have died during the preparations for the operation, or just before the arrival of the surgeon, might probably have been saved. If *during the operation* the patient sink as if lifeless, the operation must be rapidly continued, and in this way two recoveries were procured.

In this disease we should always carefully watch the patient *under the expectation of having to operate.* 1. The course of the disease is frequently excessively rapid. In more than half of 65 cases in which no operation was performed, death occurred at various periods within 24 hours. 2. Certain forms are especially remarkable for their rapid course, as those dependent on inflammation of the fauces, on anasarca after scarlatina, on cachectic diathesis ; as also when consecutive to a wound of the neck, with infiltration of blood into the cellular tissue external to the larynx. On the other hand, its progress is slower when it is dependent on deep-seated laryngeal lesions. 3. When the disease assumes the continuous form, it is much more rapid in its course than is the paroxysmal. 4. Sometimes while the patient's condition seems ameliorated, he yet dies suddenly amidst paroxysmal suffering. A remarkable calm after severe paroxysms is sometimes a precursor of death. 5. The nocturnal aggravation of the disease is indubitable, and calls for watching.

Among the varieties of *operative procedure,* the author prefers *crico-tracheotomy,* by which the difficulty often arising from an œdematous state of the neck is avoided, and the penetration of air into the veins, (this accident occurred twice in 36 operations, although no mention is made of it in 333 cases of bronchotomy for other affections,) and of blood into the air-passages, rendered less likely. It is far easier than tracheotomy, and fitter for the inexpert surgeon, called in on emergency. He gives minute directions for the performance of the operation ; but as these do not apply especially to this disease, we need do no more than refer to them.—*Ibid. from Archives Générales.*

